# Innovative therapeutic strategy using prostaglandin I_2_ agonist (ONO1301) combined with nano drug delivery system for pulmonary arterial hypertension

**DOI:** 10.1038/s41598-021-86781-3

**Published:** 2021-03-31

**Authors:** Tomomitsu Kanaya, Shigeru Miyagawa, Takuji Kawamura, Yoshiki Sakai, Kenta Masada, Nobutoshi Nawa, Hidekazu Ishida, Jun Narita, Koichi Toda, Toru Kuratani, Yoshiki Sawa

**Affiliations:** 1 Department of Cardiovascular Surgery, Graduate School of Medicine, Osaka University, 2-2 Yamadaoka Suita, Osaka, 565-0871 Japan; 2 Department of Pediatrics, Graduate School of Medicine, Osaka University, Osaka, Japan

**Keywords:** Hepatocyte growth factor, Drug delivery, Respiration

## Abstract

Clinical outcomes of pulmonary arterial hypertension (PAH) may be improved using targeted delivery system. We investigated the efficacy of ONO1301 (prostacyclin agonist) nanospheres (ONONS) in Sugen5416/hypoxia rat models of PAH. The rats were injected with saline (control) or ONONS (n = 10, each) on days 21 and 28, respectively. Hepatocyte growth factor (HGF)-expressing fibroblasts and inflammatory cytokines were measured. Cardiac performance was assessed and targeted delivery was monitored in vivo, using Texas red-labeled nanoparticles. Compared with control, HGF-expressing fibroblasts and HGF expression levels were significantly higher in the ONONS group, while the levels of interleukin-6, interleukin-1β, transforming growth factor-β, and platelet-derived growth factor were lower. Histological assessment revealed significant amelioration of the percent medial wall thickness in pulmonary vasculature of rats in the ONONS group. Rats in the ONONS group showed decreased proliferating cell nuclear antigen-positive smooth muscle cells and improved right ventricle pressure/left ventricle pressure. No difference was seen in the accumulation of Texas red-labeled nanoparticles in the brain, heart, liver, and spleen between PAH and normal rats. However, a significant area of nanoparticles was detected in the lungs of PAH rats. ONONS effectively ameliorated PAH, with selective delivery to the damaged lung.

## Introduction

Idiopathic pulmonary artery hypertension (IPAH) is a progressive disease characterized by an elevation of pulmonary vascular resistance, ultimately resulting in right ventricular failure and death. It has been estimated that the 5-year mortality rate of IPAH is 20 to 30%^1–4^. Intravenous prostacyclin agonist works well, but has some adverse effects, and it is not convenient. Thus, there is a need to identify alternative treatment approaches, using innovative concepts, to restore the functionality of impaired lungs in IPAH.

ONO1301 is a molecule that exerts long-lasting prostacyclin activity via its inhibitory effect on thromboxane synthase. It is chemically and biologically stable owing to its nonprostanoid structure^5,6^. ONO1301 has a high binding affinity for prostacyclin receptors (IPR). Stimulation of the IPR by ONO1301 promotes cAMP elevation, leading to the upregulation of the expression of hepatic growth factor (HGF), vascular endothelial growth factor (VEGF), and stromal cell-derived factor 1 (SDF-1), both in vitro and in vivo^5^. A study reported that HGF significantly mitigated the severity of PAH and inhibited inflammation in the lungs of PAH rats^7^. Thus, ONO1301 is expected to play a role as a new therapeutic agent for PAH.

Although ONO1301 has been developed as antiplatelet drug, Phase 1 clinical trial showed side effects such as diarrhea and headache. Therefore, there is a need to develop targeted-drug delivery to the damaged tissue so as to minimize side effects. As organ-targeted therapy, various nano drug delivery systems have been investigated, especially for the delivery of anti-cancer drugs^8,9^. These systems enable the delivery of drug-loaded nano particles, containing minimal dose of the drug, specifically to the damaged tissue. In addition, these nanoparticles have enhanced permeaability across the blood vessels^10,11^.

In this study, we investigated whether systemic administration of ONO1301, using nano drug system, can ameliorate pulmonary artery hypertension (PAH), and thereby result in an improvement of hemodynamic condition in Sugen/hypoxia rat model of PAH.

## Results

### Protein levels of HGF in normal human lung fibroblast

**Figure 1:**
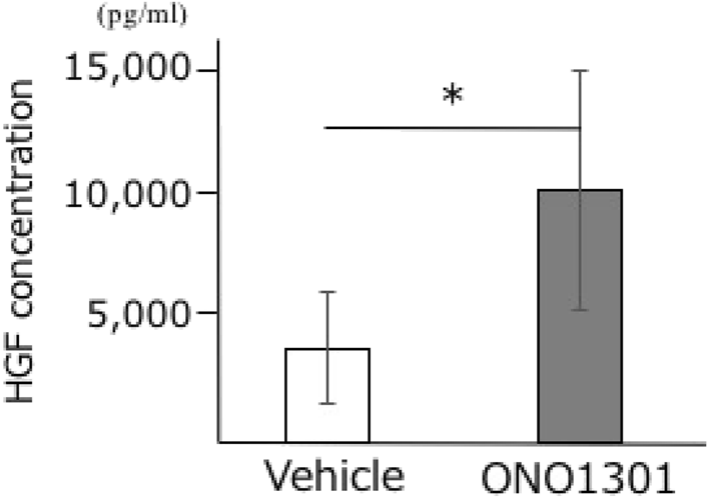
ONO1301 promotes the production of hepatocyte growth factor (HGF) in normal human lung fibroblast (NHLF). Exposure to ONO1301 (100 nM) led to a significant increase in the secretion of HGF in the culture supernatant of NHLF at 72 h, as measured by ELISA. n = 5. *P < 0.05.

The protein levels of HGF in normal human lung fibroblast (NHLF) were measured using the ELISA technique. Results showed that treatment with ONO1301 led to a significant increase in the HGF levels in the supernatant of the cultured NHLF at 72 h (Fig. 1).

### Anti-proliferative effects of HGF on pulmonary smooth muscle cells

**Figure 2:**
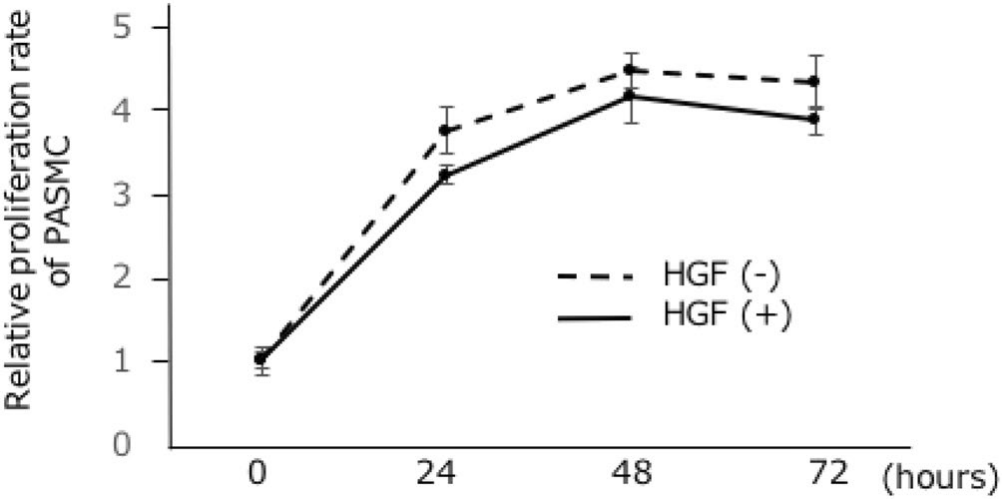
Proliferation assay of pulmonary smooth muscle cells (PASMC) after treatment with hepatocyte growth factor (HGF). HGF treatment (10 ng/mL) significantly suppressed the proliferation of PASMCs at 24, 48, and 72 h, respectively. n = 5. *P < 0.05.

Effects of HGF on the proliferation of pulmonary smooth muscle cells (PASMCs) were assessed in vitro. HGF treatment (10 ng/mL) significantly suppressed the proliferation of PASMCs at 24, 48, and 72 h, respectively (Fig. 2).

### Desmosomes between endothelial cells in the lungs of PAH rats

**Figure 3:**
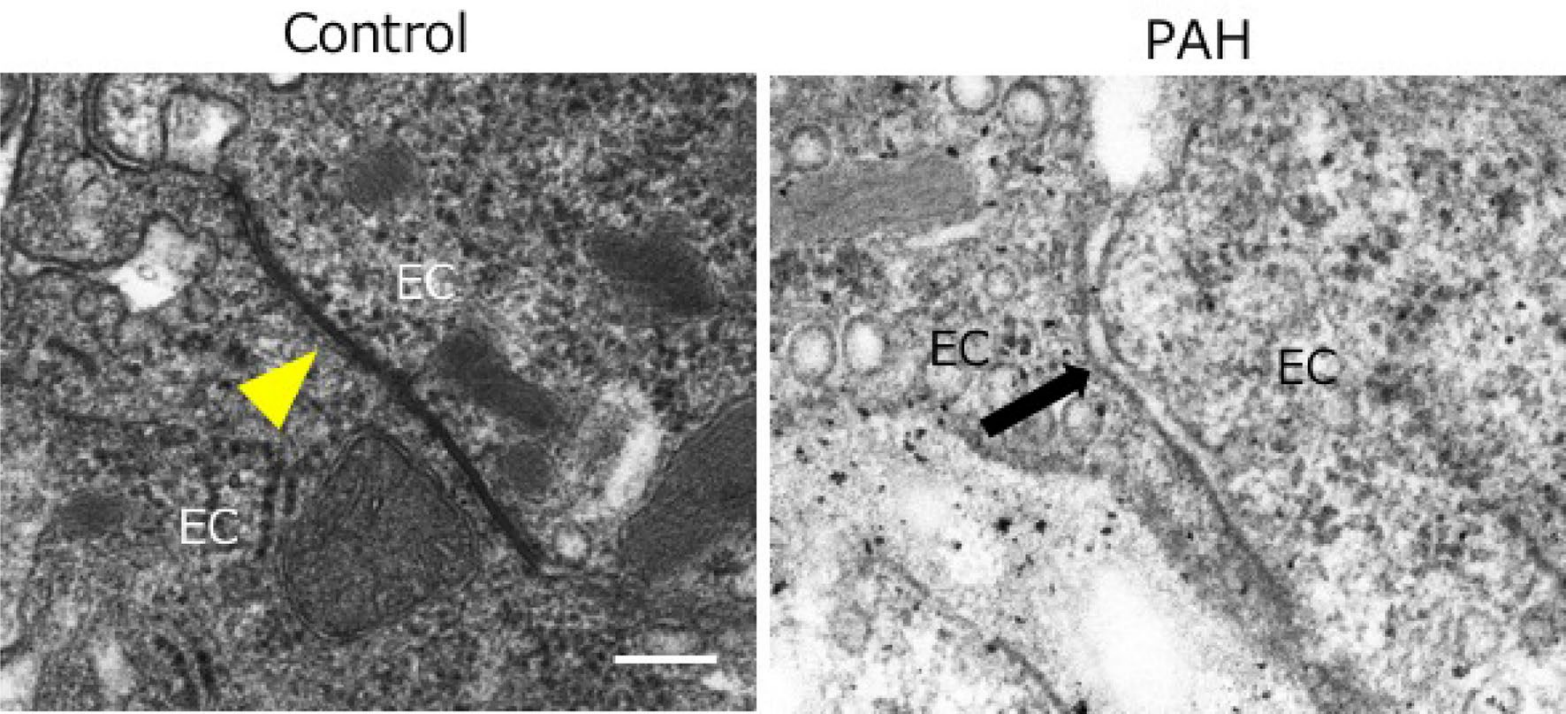
Presence of desmosome between endothelial cells in normal rat and pulmonary hypertension rat. Electronic microscopy showed the presence of desmosome between endothelial cells in control rat (yellow arrow). However, no desmosome was identified in pulmonary hypertension rat (black allow) at 35 days after subcutaneous injection of Sugen. Bar = 200 nm. *EC* endothelial cell.

We confirmed intercellular junctions of endothelial cells using Electron microscopy. In normal rat lung tissue, the small disk-shaped adhesive junction with a size of 200 to 500 nm, the gap between the opposing endothelial cell membranes with 22 to 35 nm, and a fine fiber structure can be seen in this gap. These are the characterization of “desmosome”. However, in PAH lung tissue, almost no desmosome between endothelial cells was observed (Fig. 3). These results indicated that the disrupted endothelial integrity.

### Permeability of pulmonary vessels in PAH rat

**Figure 4:**
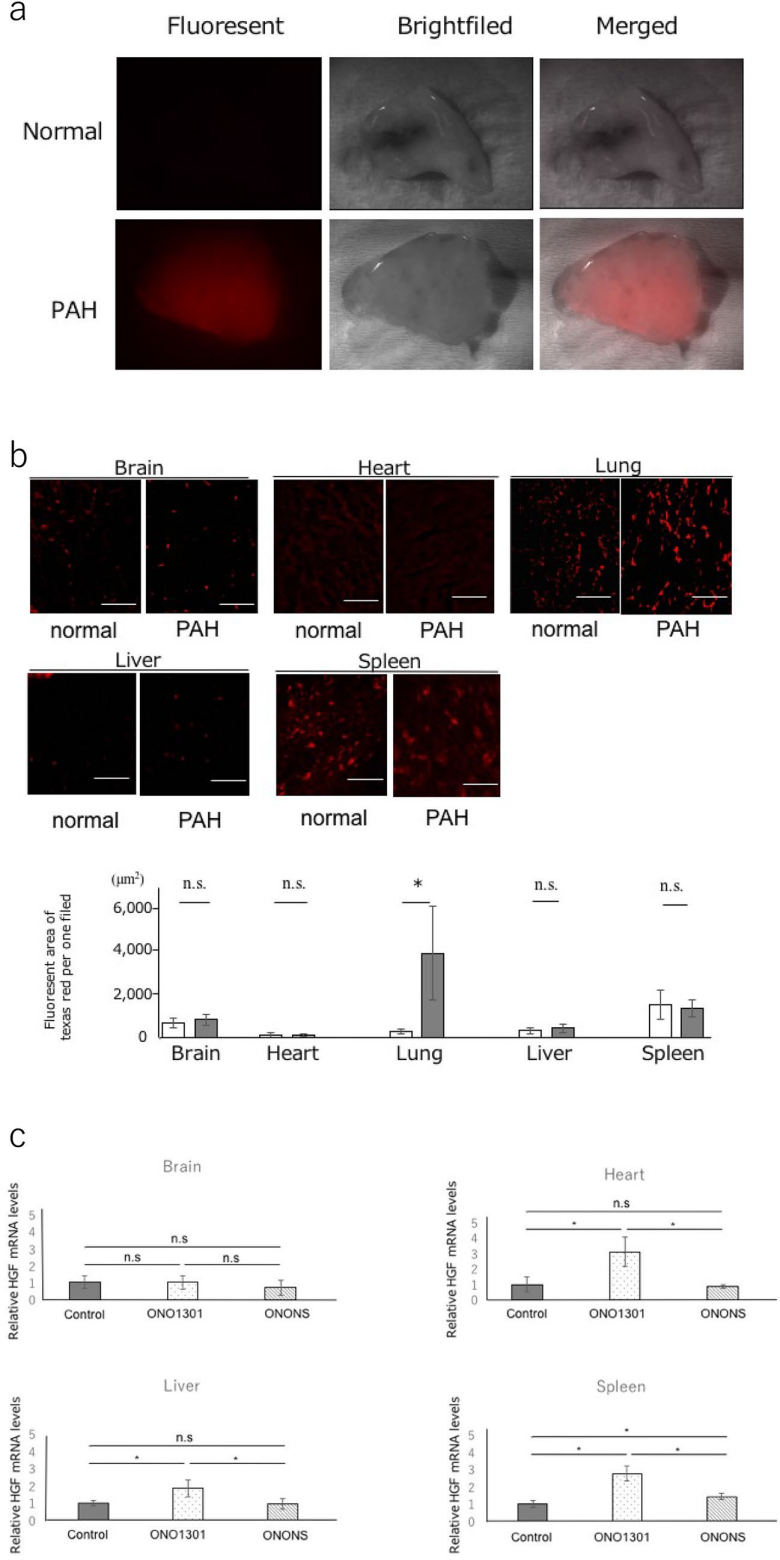
Permeability of the PAH lung and tissue distribution of nano particles. (**a**) Fluorescent comparison in vascular permeability between control and pulmonary artery hypertension (PAH) rat. The representative lung images of control rat and PAH rat which was injected fluorescent-labeled nano-sized beads (100 nm) are shown in the upper and lower panels, respectively. The lungs were excised 15 min after the injection of fluorescent nano-sized beads. (**b**) Tissue distribution of Texas red in each organ. The accumulation of Texas red was significantly elevated in the lungs of PAH rats compared with control rat (n = 5). There was no significant difference in other organs between control rats and PAH rats (n = 5). Bar = 50 μm. The upper panels show representative images of the the Texas red staining of the lung in normal and PAH rats. The lower panels show corresponding quantifications. *P < 0.05. *n.s.* not significant. (**c**) Comparison of the effect on each organ in the PAH rats treated with ONO1301 and ONONS. HGF mRNA levels were measured on the brain, heart, liver, spleen in the control group, ONO1301 group, and ONONS group. Except for the brain, the expression levels of HGF are significantly higher in ONO1301 group than in ONONS group (n = 5). While, in the ONONS group, HGF expression levels are not significantly high compared to the control group. *P < 0.05. *n.s.* not significant.

We assessed the pulmonary vascular permeability in normal and PAH rats, using nano-sized fluorescent beads (100 nm). Ex vivo fluorescent image analysis showed an accumulation of nano-sized fluorescent beads in the lungs of PAH rats, 15 min after the injection of the fluorescent beads. However, the lungs of normal rats did not show the presence of fluorescent beads (Fig. 4a).

### Tissue distribution of nano particles labeled with Texas red

To evaluate the tissue distribution of nanoparticles, we performed immunohistochemical analysis using nano particles labeled with Texas red. After injection of nano particles labeled with Texas red, no significant difference was observed between the brain, heart, liver, kidney, and spleen of normal rats and PAH rats. However, there was a significant difference between the lungs of normal rat and that of PAH rat (80.5 ± 30.8 vs 3751.6 ± 2208 μm^2^; p < 0.01) (Figs. 4b).

### Effect for ONONS on other tissues

To confirm the differences of the effect of ONO1301 and ONONS on the other tissues in PAH rats, we examined the mRNA levels of HGF, as a marker of ONO1301, and compared these with the control group, only ONO1301 group, and ONONS group (n = 5). The gene expressions of HGF in the heart, liver, and spleen were significantly higher in the ONO1301 group than in ONONS (Fig. 4c). It is possible to have the action for the normal tissue in the ONO1301 group and similarly, to have less effect on other organs in ONONS group.

### Uptake of nano particles-labeled Texas red in the fibroblasts

**Figure 5:**
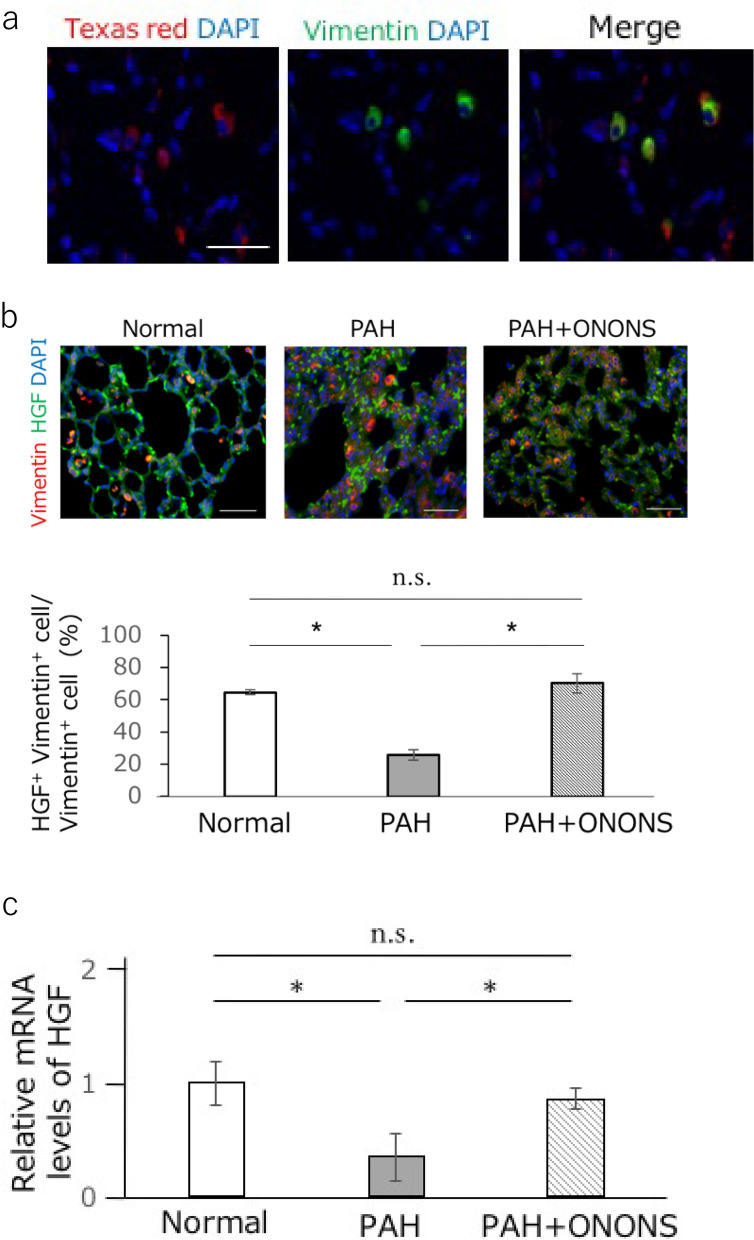
The expression of hepatocyte growth factor (HGF) in vimentin positive cells and HGF expression in the lung of pulmonary artery hypertension (PAH) rat with ONONS treatment. (**a**) Nano particles injected intravenously were taken up by fibroblasts in the lungs of PAH rats. Nano particles labeled with Texas red were stained for vimentin positive cells. Bar = 20 μm. (**b**,**c**) HGF-expression vimentin positive cells (yellowish cells) were significantly increased in ONONS group. The upper panels show representative images and the lower panel is the corresponding quantification. Bar = 20 μm. n = 5. *P < 0.05. (**d**) The expression level of HGF in the lung was significantly increased in ONONS group. n = 5. *P < 0.05.

We determined the target cells of ONONS in the lungs using immunohistochemical analysis. Results showed that while vimentin positive cells were double stained with Texas red by immunohistochemical analysis, CD31 positive cells and αSMA positive cells were not double stained (Fig. 5a).

### Increased HGF in the lungs of PAH rats treated with ONO1301

Fibroblasts which were stained by vimentin were double stained by HGF (HGF^+^ fibroblast). In addition, ONONS treatment significantly increased HGF^+^ fibroblast levels and upregulated the expression of HGF in the lung tissue (Fig. 5b,c).

### Proliferation of pulmonary smooth muscle cells in the lungs of PAH rats

**Figure 6:**
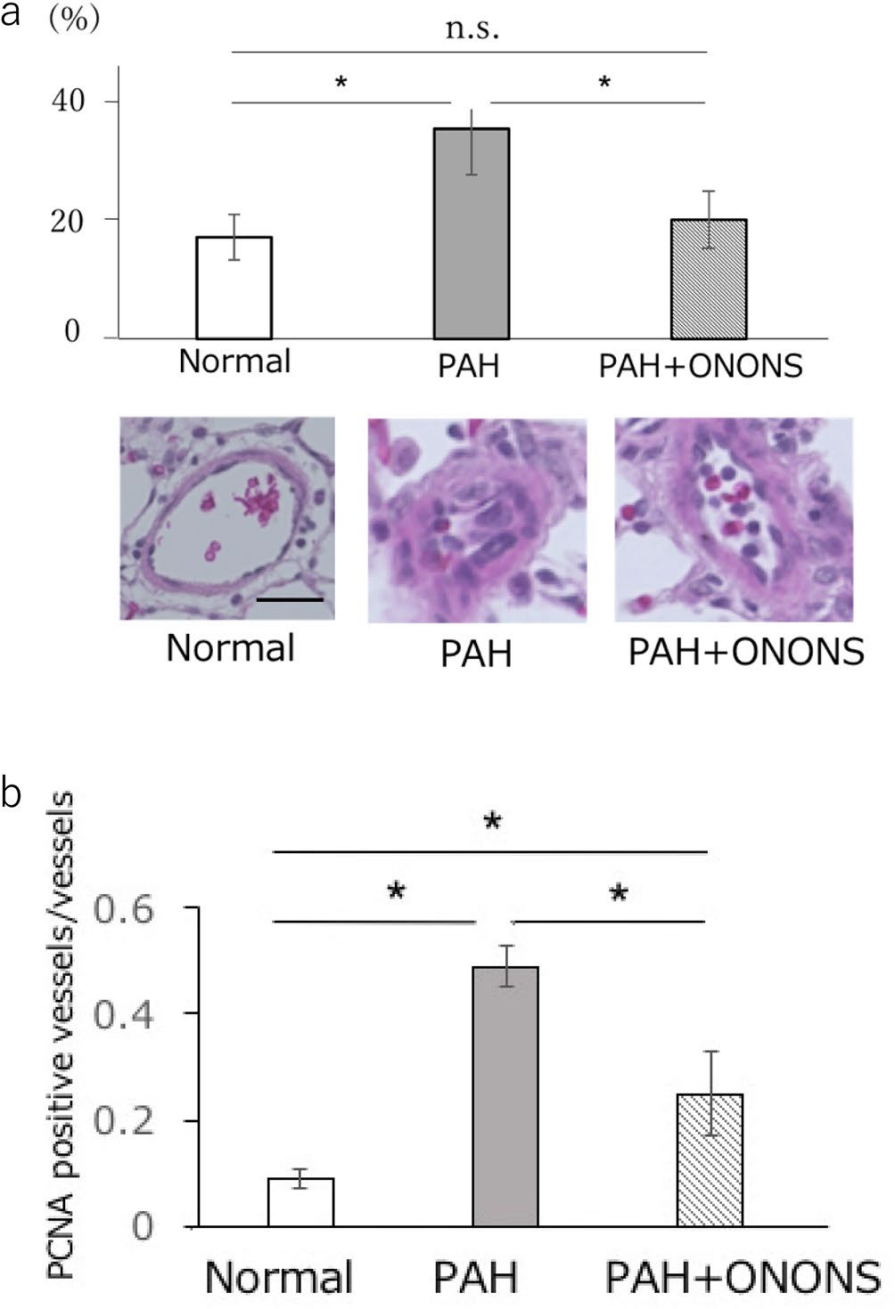
Histological analysis of proliferation of smooth muscle cells proliferation in PAH rats treated with ONO1301 nanospehre (ONONS). (**a**) The percent medial wall thickness in pulmonary small arteries was significantly decreased in the ONONS rats. (**b**) Proliferating cell nuclear antigen (PCNA) positive smooth muscle cells were significantly decreased in ONONS treated rats. n = 10. *P < 0.05.

To evaluate the proliferation of smooth muscle, we performed immunohistological analysis of small pulmonary artery. In the ONONS group, the numbers of PCNA positive smooth muscle cells significantly decreased group (0.49 ± 0.04 vs 0.25 ± 0.09 /vessel; p < 0.05) and the percent medial wall thickness in pulmonary vasculature was significantly enhanced (36.6 ± 8.5 vs 20.8 ± 4.8%; p < 0.01). In addition, we observed that the proliferation of smooth muscles in the small pulmonary artery was decreased in the ONONS group (Fig. 6a,b).

### Effects of ONO1301 on inflammatory markers in the lungs

**Figure 7:**
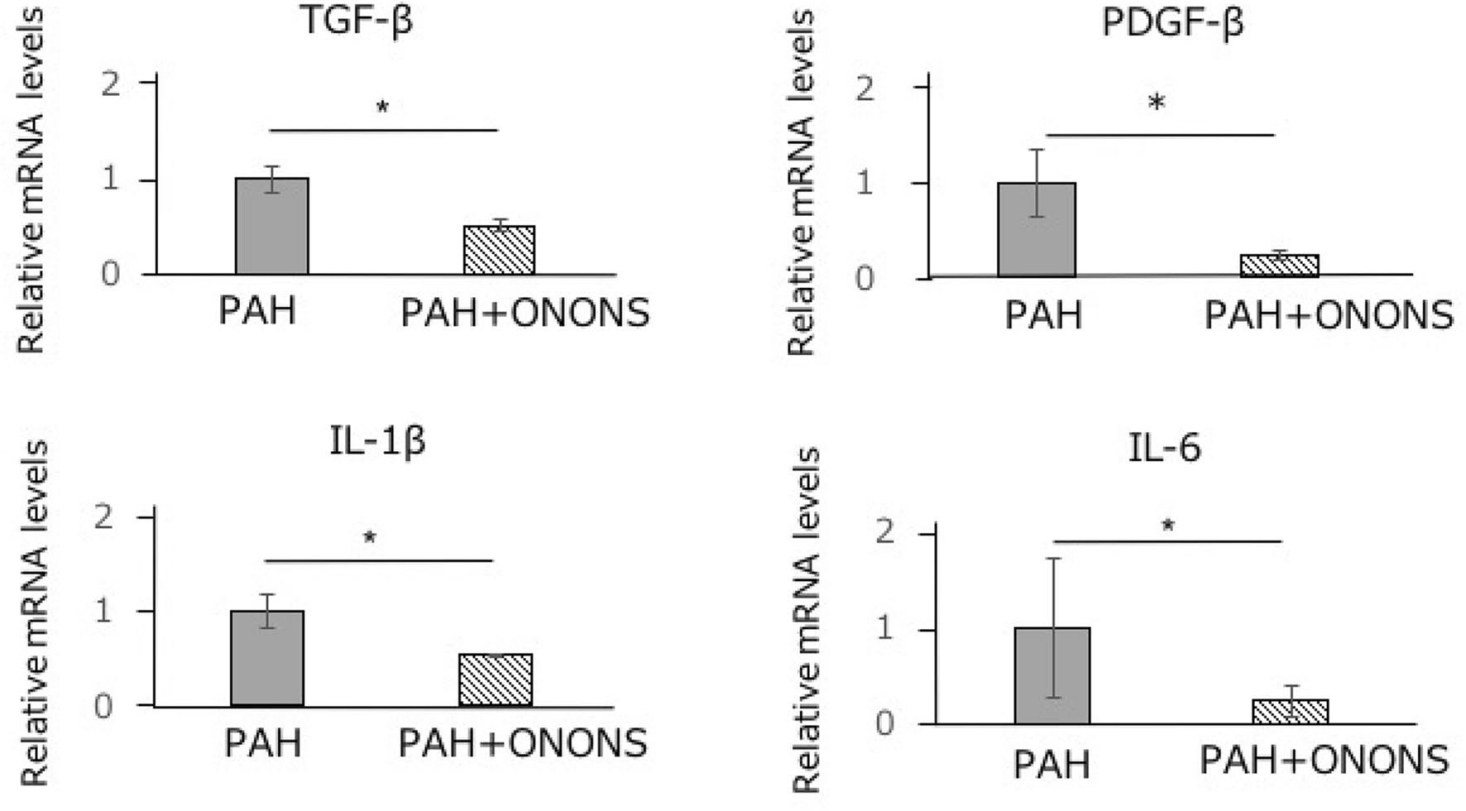
Effect of ONO1301 on the production of inflammatory markers. ONO1301 nanosphere (ONONS) treatment significantly suppressed expression of TGF-β, PDGF-β, IL-1β, and IL-6. *P < 0.05. n = 5. *n.s.* not significant.

To confirm inflammation in the lungs of PAH rats, we examined the mRNA levels of inflammatory markers, including interleukin -1β (IL-1β) and interleukin -6 (IL-6). The gene expression of IL-1β and IL-6 was significantly lower in the ONONS group than in the control group. This indicated that ONO1301 had anti-inflammatory effect. To evaluate smooth muscle proliferative effect of ONO1301 in the lungs of PAH rats, we examined the mRNA levels of inflammatory markers, including transforming growth factor (TGF-β)and platelet derived growth factor-β (PDGF-β). The gene expression of TGF-βand PDGF-βwas significantly reduced in the ONONS group than in the control group (Fig. 7).

### Hemodynamic assessment and right ventricular remodeling

**Figure 8:**
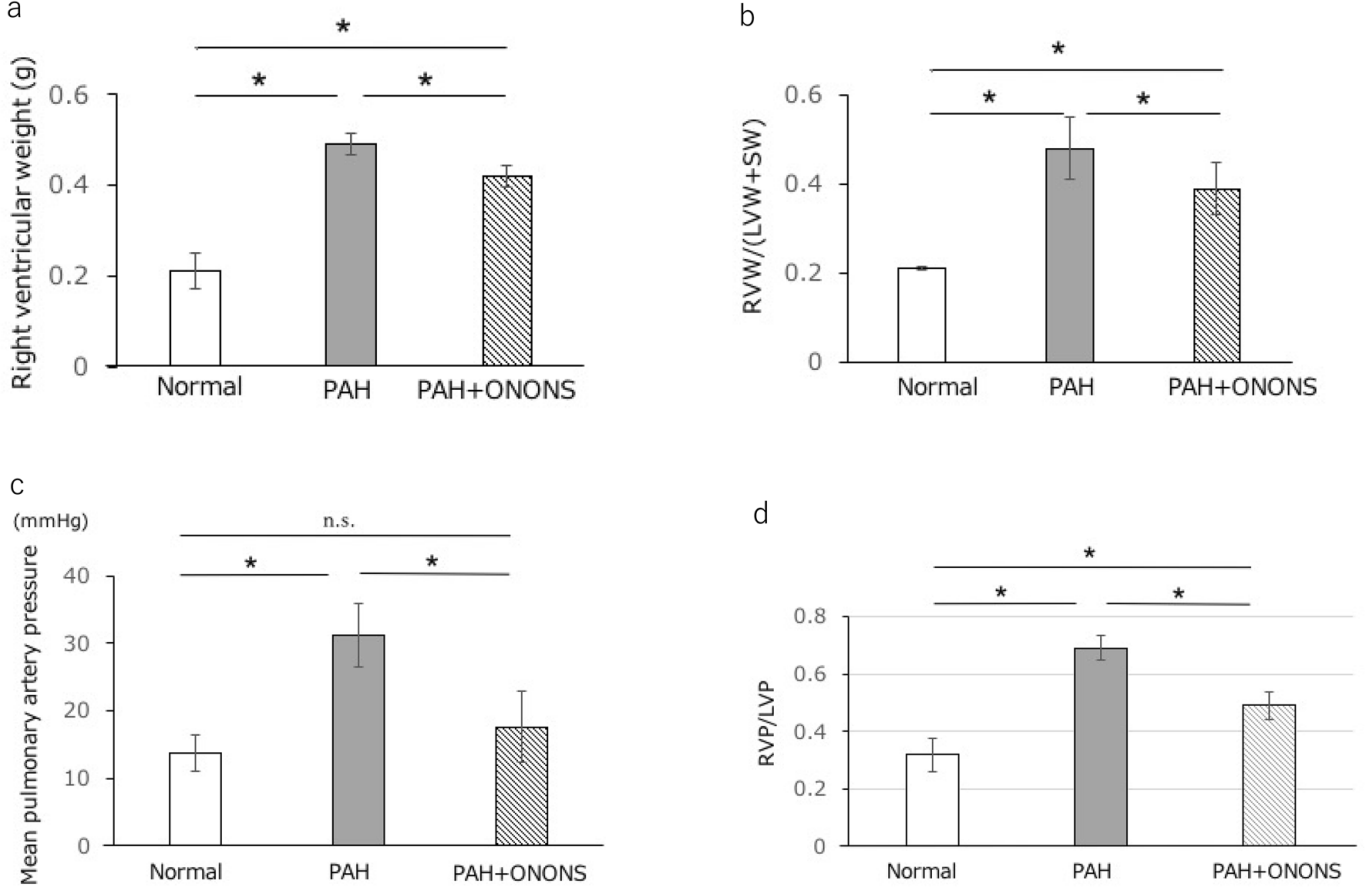
Hemodynamic effects of ONO1301 nanospehre (ONONS) in pulmonary artery hypertension (PAH) rats. (**a**) Right ventricle weight. (**b**) The weight ratio of the right ventricle to the left ventricle and septum (RVW/LVW + SW). (**c**) Mean pulmonary artery pressure. (**d**) The pressure ratio of right ventricle to left ventricle. These date showed that ONONS attenuated pulmonary hypertension in PAH rats. *P < 0.05. n = 10.

Hemodynamic measurements after 35 days of SU5416 injection revealed that the mean pulmonary artery pressure was significantly lower in the ONONS group (17.6 ± 5.2 vs 31.2 ± 4.6 mmHg; p < 0.05). In addition, a significant reduction in right ventricle pressure to left ventricle pressure ratio (RVP/LVP ratio) was observed in the ONONS group (0.49 ± 0.12 vs 0.68 ± 0.11; p < 0.05). The right ventricle weight to left ventricle weight and septal wall weight ratio (RVW/(LVW + SW) in the ONONS group was significantly lower (0.39 ± 0.07 vs 0.49 ± 0.07; p = 0.02). These results indicated that pulmonary hypertension and right ventricle hypertrophy were improved after treatment with ONONS (Fig. 8).

## Discussion

In the present study, ONO1301 acted on lung fibroblasts, leading to an elevation of HGF, decrease in inflammatory cytokines, and a suppressive effect on lung vascular smooth muscle proliferation in the lungs of PAH rats. Systemic administration of ONO1301, using nano drug system, specifically ameliorated PAH in rats (induced by Sugen/hypoxia), and improved hemodynamic condition, which led to the control of the progression of pulmonary hypertension.

The PAH lung is characterized by inflammatory condition, with elevated TGF-β, PDGF-β, and inflammatory cytokines, including IL-6 and IL-1β^12–14^. These inflammatory cytokines induce the proliferation and migration of vascular endothelial cells and smooth muscle cells, resulting in vascular remodeling. Endothelial dysfunction leads to a decrease in endogenous prostacyclin and cyclic adenosine monophosphate (cAMP), resulting in impaired diastolic dysfunction of the vascular smooth muscle, which is also considered to cause exacerbation of PAH^15^.

ONO1301 has been reported to have vasodilatory effect and antiplatelet aggregating activity through prostacyclin receptor (IPR)^16^. Furthermore, studies have shown that ONO1301 inhibits extra cellular signal-regulated protein kinease (ERK) phosphorylation related to PDGF, and promotes HGF secretion, acompanied with TGF-β suppression^17,18^. However, there is no report describing the cell-specific targeted effects of ONO1301 and the underlying mechanism. In this study, we demonstrated that ONO acts primarily on the lung fibroblasts, promotes HGF secretion, and subequently suppresses pulmonary vascular muscle cells proliferation, both in vitro and in vivo. The increase in HGF leveles in turn supppressed inflammatory cytokines, including TGF-β, IL-1β, and IL-6, and these anti-inflammatory effects of HGF ultimately improved pulmonary hypertension. Previous studies reported that ONO1301 suppressed ERK phosphorylation, leading to inhibition of PDGF, with suppression of remodeling vascular obstructive lesions. Although the direct action of ONO1301 as an IPR agonist was not investigated in this study, ONO1301 has a potential for increasing cAMP in the smooth muscle cells and improving diastolic dysfunction, resulting in reduction of pulmonary hypertension. Recently, it has been reported that miRNAs represent disease pathophysiology or propose new treatment targeting to miRNA. One of the therapeutic mechanisms proposed for ONO1301 include its ability to induce exosomes, including several cytokines and miRNAs from endothelial cells^19^.

In the present study, ONO1301 acted on pulmonary fibroblasts to promote HGF secretion, leading to anti-inflammatory effect and proliferation inhibitory effects on the pulmonary vascular smooth muscle cells. These effects attenuated PAH and right ventricular hypertrophy in Sugen/hypoxia model rats.

Nanoparticles have been used in numerous novel delivery systems for the transport of drugs to the target organs. Nanoparticles are taken up by the target organ owing to their small size, which allows them to permeate into tissue and be retained. Nanoparticles for specific delivery can optimize the efficacy and minimize the side effects of drugs. The use of a nano-drug delivery system can enhance the efficacy and safety of therapeutic agents, and may overcome drawbacks such as toxicity, low water solubility, and poor bioavailability^8^.

Although drug therapy using nano-drug delivery system for PAH have been described previously^9,20^, the effect of drug delivery system on other organs has not been clearly elucidated. This study revealed disruption in endothelial integrity via electron microscopy and elevated vascular permeability of the lungs of PAH rats, via fluorescent image analysis. Additionally, immunohistological analysis showed that while no significant uptake of nanoparticles was observed in the brain, heart, liver, and spleen of PH rat compared with normal rat, a significant accumulation of the nanoparticles was observed in the lung of PH rat. Therefore, it was evident that nano drug delivery system of ONO1301 allowed selective accumulation of the drug in the PAH lung, without affecting other healthy organs and disruption of vasculature, thus indicating that the dose of drug can be minimized, without compromising on efficacy.

In conclusion, intra venous infusion of ONONS with targeted delivery to damaged lung was effective in treating PAH, as determined by hemodynamical and histological analysis of PAH in rats, these results suggest that nano drug delivery formulation of ONO1301 may be used as an innovative drug delivery strategy for the treatment of PAH.

## Methods

All experimental protocols, including in vitro and in vivo studies, were approved by the Ethics Committee and Animal Experimental Faculty of Osaka University Graduate School of Medicine. We confirmed that all methods were carried out in accordance with relevant guidelines and regulations. The study was carried out in compliance with the ARRIVE guideline.

### Tissue preparation

After the hemodynamic examinations, the rats were sacrificed, and the left and right lungs were excised. Both lungs were washed with phosphate-buffered saline (PBS) through a pulmonary artery cannula, and were distended and fixed by perfusion through tracheal cannulation with 4% paraformaldehyde/10% buffered formalin, under a constant pressure of 20 mmHg^21^. After ligation of the trachea, the lungs were fixed by immersion in 4% paraformaldehyde/10% buffered formalin overnight. They were then blocked and embedded in paraffin and cut into 5 μm thick sections^22,23^.

### Histological analysis

The pulmonary arteries were stained with Elastica Van Gieson stain and percent medial wall thickness was calculated in ten muscular arteries of resistant arteries (outer diameter of 50–200 μm) per lung, using the following formula: 100 × (external diameter – internal diameter)/external diameter^23^.

### Immunocytochemistry

The 5-μm serial coronal sections of the lungs of PAH rats were deparaffinized and washed. Epitope retrieval was performed using Target Retrieval Solution (Dako, Gostrup, Denmark). The sections were then washed with PBS and nonspecific binding was blocked with Protein Block, Serum Free (Dako). Primary antibodies targeting the following proteins were used for immunohistochemistry: α-smooth muscle actin (αSMA, 1:50, mouse, Abcam, Cambridge, UK), proliferating cell nuclear antigen (PCNA, 1:100, mouse, Abcam), HGF (1:50, rabbit, Abcam), vimentin (1:100, rabbit, Abcam), and Texas red (1:200, rabbit, Abcam).

The primary antibody staining was visualized using fluorescence-conjugated secondary antibodies (Life Technologies, Grand Island, NY, USA). Haechst33342 (Dojindo, Kumamoto, Japan) as used after incubation with the secondary antibodies, for nuclear counterstaining^21^.

### Primary culture of pulmonary arterial smooth muscle cells

Pulmonary arterial smooth muscle cells were cultured using the following six steps: (a) Isolation of the pulmonary artery from the pulmonary hypertension rat, (b) removal of the fat tissue around the pulmonary artery, (c) cutting of the pulmonary artery longitudinally, (d) scraping the intima softly to eliminate endothelial cell, (e) transferring the tissue blocks to cell culture plates, and (f) incubation until the cells reach confluence. The cells were identified as PASMCs by morphology and immunofluorescence.

### Cell culture and treatment

Primary cultured PASMCs and NHLFs (Lonza, Bazel, Switzerland) were grown to confluence using Dulbecco's Modified Eagle Medium (Lonza, Bazel, Switzerland). The medium was supplemented with 10% fetal bovine serum, and cells were incubated at 37 °C, 5.0% CO_2_.

### Cell proliferation assay

Cell Counting Kit-8 (Dojindo, Kumamoto, Japan) was used for in vitro cell proliferation assay, as described previously^24^. PASMCs (3 × 10^3^ cells/well) were seeded into 96-well flat bottomed plated in 100 μL of complete medium. PASMCs were treated with HGF (10 ng/mL) and were incubated for 24, 48, and 72 h. Cholecystokinin solution (10 μL) was added to each well, and the cells were incubated for an additional 2 h. Absorbance was measured at 450 nm using a microplate reader. Three independent experiments were performed in triplicate.

### Enzyme-linked immune sorbent assay (ELISA)

The concentration of HGF in culture supernatant of NHLF was determined by ELISA (R&D systems, Minneapolis, MN, USA), according to the manufacturers’ instructions. ONO1301 (100 nM) was added to each well, containing 1.0 × 10^6^ cells/dish and incubated for 72 h, before evaluation. Individual in vivo or in vitro samples were tested in duplicate and the concentrations of tissue HGF were calculated by the standard curve, established using recombinant individual HGF provided.

### Quantitative real-time polymerase chain reaction analysis

Total RNA was extracted from the lungs of the rats (n = 5 per group), using RNeasy Kit and reverse-transcribed, using SuperScript VILO Master Mix (Life Technologie, Carlsbad, CA, USA). Real-time PCR was performed using the 7500 Fast Real-Time PCR System (Applied Biosystems, Foster City, CA, USA), using primers for *Hgf* (Rn00566673-m1)*, Il-1β(*Rn00580432-m1*), TGFβ* (Rn00572010-m1), *PDGFβ* (Rn01502596-m1), *Il-6* (Rn01410330-m1), and *GAPDH* (Rn01775763-g1). Gene expression levels were normalized by relative expression to *GAPDH.*

### Preparation of ONONS

We used the same ONONS reported by Yajima et al.^25^. 1,2-dierucoyl-sn-glycerol-3-phosphorylcholine (1.88 g, CAS No. 5177–95-4, Nippon Fine Chemical, Osaka, Japan) and N-(carbonyl-methoxypoly-ehyleneglycol 2000)-1,2 distearoyl-sn-glycero-3-phosphosphoethanolamin sodium salt (0.12 g, CAS No.147867-65-0, Nippon Fine Chemical), were dissolved, together with ONO1301 (100 mg, Ono Pharmaceuticals, Osaka, Japan), in 20 g of t-butanol at 70 °C. The obtained solution was immediately frozen in dry ice/acetone, followed by freeze-drying for 17 h. The obtained powder was dispersed in phosphate-buffered saline in a warm bath (50 °C) and sonicated until lumps disappeared. The solution was passed through a polycardonate filter with pores of 400 nm in diameter and then trough another filter, with pores of 200 nm in diameter, to obtained a translucent liposome fluid. The obtained solution was purified by ultrafiltration to prepare consistently nanosized and round-shaped ONO1301 nano spheres (ONONS). ONO1301 nano spheres were prepared by mixing the drug and the lipid (1,2-dierucoyl-sn-glycerel-3-phosphorylcholine: N-(carbonyl-methoxypolyethyleneglycol 2000)-1,2 distearoyl-sn-glycero-3-phosphoethanolamine sodium salt = 94:6) in a drug-to-lipid ratio of 0.05. ONONS was stored at 4 °C, and took just prior to injection.

### Animal model of pulmonary arterial hypertension

The Sugen-5416/Hypoxia rat model of PAH was prepared using the established methods^26^. Male Sprague Dawley rats (Japan SLC, Shizuoka, Japan), weighing 180–220 g, were subcutaneously injected with SU5416 (20 μg/g) and exposed immediately to chronic hypoxia (depressurized to 50 kPa) for 21 days. The rats were kept in normoxic (room air) chambers for an additional 14 days until the day of the assessment. They were then randomized into the following two groups: Nano group (nano particles, containing ONO1301 (3 μg/g)) were injected intravenously via the tail vein on 21 and 28 days, n = 10) and Control group (saline was injected to PAH rats, n = 10).

### Vascular permeability in the pulmonary hypertensive lungs

The vascular permeability in the pulmonary hypertensive lungs of the PAH rats was examined. We used carboxylate-modified beads (diameter: 100 nm), labeled with red fluorescent dye (580/605; absorption/emission wavelength in nm, FluoSpheres F8801, Invitrogen, Carlsbad, CA, USA). The beads were dissolved in saline (500 μL/rat, 20% vol/vol) and administered to the rats via intravenous injection. Fifteen minutes later, ex vivo fluorescence images were obtained using fluorescence microscope (Leica M205FA fluorescence stereo microscope).

### Tissue distribution of nano particles

To examine the tissue distribution of nano particles, nano particles encapsulated Texas red (Katayama Chemical Co. Osaka, Japan) were prepared. PAH rats and normal rats were administered a single injection of the nano particles containing Texas red, via the tail vein on day 35, after the injection of SU5416. Each rat received 1.5 μL/g of the nano particles, which was the same amount of ONONS. Twenty-four hours after the injection, the rats were sacrificed for the collection of brain, heart, lung, liver, and spleen.

### Comparison of the effect on other organs in the PAH rats treated with ONO1301 and ONONS

To examine the impact of nanoparticle formulation and the confirmation for the drug delivery, the mRNA levels of HGF as one of the indicators of ONO1301 on the brain, heart, liver, spleen, were measured and compared in the PAH rat (control group), the PAH rat treated with ONO1301 (ONO1301 group), and the PAH rat treated with ONONS (ONONS group). 5 μl/g nano particle not containing ONO1301, 3 μg/g ONO1301 in 5 μl/g phosphate-buffered saline (ONO1301 group), and 3 μg/g ONO1301NS in 5 μl/kg phosphate-buffered saline (ONONS group) were injected on 21and 28 days after injection of SU5416. On 35 days, the rats were sacrificed for the collection of the brain, heart, liver, and spleen.

### Assessment of right ventricular hypertrophy

After sacrificing the rat, the right ventricle was dissected from the left ventricle plus septum (LV + S) and weighed separately. The weight ratio (RVW/(LVW + SW)) was calculated.

### Assessment of hemodynamics

Hemodynamic measurements were performed as described previously^22,27^. Hemodynamics were evaluated after 35 days of SU5416 injection. Rats were anesthetized with isoflurane and mechanically ventilated. After small left thoracotomy, right ventricular systolic pressure and left ventricular systolic pressure were measured with 23-gauge needle and pressure transducer (MLT0699; AD Instruments, Colorado Springs, CO, USA)^22,23^.

### Statistical analysis

In the two-group comparison, Student’s t-test or the X^2^ test was performed. Multiple comparisons were evaluated by a one-way ANOVA with Tukey’s honest significant difference test. All *p* values are two-sided, and values of *p* < 0.05 were considered statistically significant. Continuous variables are presented as mean ± standard deviations (SD). The statistical analyses were performed using JMP 12.2.0 (SAS Institute, Cary, NC, USA).
